# Oral Diabetes Medication Videos on Douyin: Analysis of Information Quality and User Comment Attitudes

**DOI:** 10.2196/57720

**Published:** 2024-10-18

**Authors:** Baolu Zhang, Surintorn Kalampakorn, Arpaporn Powwattana, Jutatip Sillabutra, Gang Liu

**Affiliations:** 1 Department of Nursing The Affiliated Hospital Southwest Medical University Luzhou China; 2 School of Nursing Southwest Medical University Luzhou China; 3 Department of Public Health Nursing Faculty of Public Health Mahidol University Bangkok Thailand; 4 Department of Biostatistics Faculty of Public Health Mahidol University Bangkok Thailand; 5 Department of Orthopedics and Center for Orthopedic Diseases Research The Traditional Chinese Medicine Affiliated Hospital Southwest Medical University Luzhou China

**Keywords:** diabetes, oral diabetes medication, information quality, user comment attitude, video analysis, Douyin

## Abstract

**Background:**

Oral diabetes medications are important for glucose management in people with diabetes. Although there are many health-related videos on Douyin (the Chinese version of TikTok), the quality of information and the effects on user comment attitudes are unclear.

**Objective:**

The purpose of this study was to analyze the quality of information and user comment attitudes related to oral diabetes medication videos on Douyin.

**Methods:**

The key phrase “oral diabetes medications” was used to search Douyin on July 24, 2023, and the final samples included 138 videos. The basic information in the videos and the content of user comments were captured using Python. Each video was assigned a sentiment category based on the predominant positive, neutral, or negative attitude, as analyzed using the Weiciyun website. Two independent raters assessed the video content and information quality using the DISCERN (a tool for assessing health information quality) and PEMAT-A/V (Patient Education Materials Assessment Tool for Audiovisual Materials) instruments.

**Results:**

Doctors were the main source of the videos (136/138, 98.6%). The overall information quality of the videos was acceptable (median 3, IQR 1). Videos on Douyin showed relatively high understandability (median 75%, IQR 16.6%) but poor actionability (median 66.7%, IQR 48%). Most content on oral diabetes medications on Douyin related to the mechanism of action (75/138, 54.3%), precautions (70/138, 50.7%), and advantages (68/138, 49.3%), with limited content on indications (19/138, 13.8%) and contraindications (14/138, 10.1%). It was found that 10.1% (14/138) of the videos contained misinformation, of which 50% (7/14) were about the method of administration. Regarding user comment attitudes, the majority of videos garnered positive comments (81/138, 58.7%), followed by neutral comments (46/138, 33.3%) and negative comments (11/138, 8%). Multinomial logistic regression revealed 2 factors influencing a positive attitude: user comment count (adjusted odds ratio [OR] 1.00, 95% CI 1.00-1.00; *P*=.02) and information quality of treatment choices (adjusted OR 1.49, 95% CI 1.09-2.04; *P*=.01).

**Conclusions:**

Despite most videos on Douyin being posted by doctors, with generally acceptable information quality and positive user comment attitudes, some content inaccuracies and poor actionability remain. Users show more positive attitudes toward videos with high-quality information about treatment choices. This study suggests that health care providers should ensure the accuracy and actionability of video content, enhance the information quality of treatment choices of oral diabetes medications to foster positive user attitudes, help users access accurate health information, and promote medication adherence.

## Introduction

Diabetes has emerged as a major public health challenge worldwide. As of 2021, around 537 million adults worldwide were diagnosed with diabetes, with projections indicating an increase to 783 million by 2045 [1]. China has the largest number of people with diabetes, with 140 million people constituting 26.2% of the worldwide diabetes population [1]. Notably, 96% of these cases are of type 2 diabetes mellitus (T2DM) [2]. A study conducted between 2007 and 2017 in China revealed an increase in diabetes awareness from 39.4% to 53.6%. However, the rates of treatment and glycemic control were suboptimal, standing at 32.5% and 32.8%, respectively [3]. Effective glycemic control is crucial in preventing diabetes-related microvascular and macrovascular complications [4]. Therefore, it is imperative to take action to enhance glycemic control in patients with diabetes.

Pharmacotherapy, including insulin therapy and oral medications, remains the predominant method of glycemic control, in addition to lifestyle modifications [5]. Research indicates that oral medications are more effective in helping patients, particularly those with diabetes who have a disease duration shorter than 5 years, achieve and maintain stable blood glucose levels compared to insulin therapy [6]. Although oral medications are crucial, nonadherence to medications remains a significant challenge. Medication nonadherence rates among patients with T2DM range from 21.2% to 59.8% [7-9]. This issue may be linked to limited health literacy among patients [10]. Improving health literacy through health education is essential for addressing this problem [11]. The provision of medication information is recognized as a fundamental aspect of diabetes health education [12]. Research indicates that 56.7% of patients with diabetes express information needs regarding oral medications, ranking second after glycemic control–related information [13]. Nevertheless, patients usually encounter difficulty in finding medical information related to their condition that is easy to understand [12]. This challenge highlights the need for more accessible and engaging forms of health education.

In recent years, social media has emerged as a promising platform for delivering health information to the public [14]. Among various social media platforms in China, Douyin (the Chinese version of TikTok), which is dedicated to the creation and sharing of short videos, is increasingly being used by doctors to publish public health education clips and is attracting many users searching for health information. Despite some differences in visual effects, privacy settings, the market, and government regulation, TikTok and Douyin are, at face value, the same [15]. The latest data report on Douyin's scientific outreach [16] reveals that content related to health care is among the most viewed by its users. By 2022, the platform hosted 35,000 certified doctors, producing over 4.43 million scientific educational content videos. Notably, diabetes ranks as 1 of the most frequently searched diseases.

Nevertheless, the informational quality of Douyin's short videos varies significantly, leading to the proliferation of misinformation. Evidence suggests that although qualified organizations and other individual users, such as health professionals and science communicators, provide information based on evidence, the material provided by for-profit firms is of poorer quality [17-19]. The presence of misinformation in health-related content on social media platforms is a growing concern, as it could potentially lead to inaccurate guidance or even health risks for users [20]. Although a study indicated that the quality of diabetes-related videos on Douyin is generally acceptable, with a primary focus on disease management, some aspects, such as the quality and comprehensiveness of information regarding oral diabetes medications, are not adequately covered, and the existing health information still does not fully meet the needs of patients [17]. Given that oral medication is a crucial component of diabetes self-management [21], assessing the quality of information and identifying potential misinformation in videos related to oral diabetes medications become imperative to meet the needs and preferences of patients and users and to ensure the dissemination of accurate health information on these platforms.

Although, like other video-sharing social platforms, the number of likes on TikTok (English version of Douyin) is an important indicator of user preferences [22], user comments, as a vital channel for interaction and communication with creators, more effectively express user views and emotional attitudes, as they encompass text, emojis, and other forms of expression [23]. Current research focusing on user preferences in video content predominantly uses likes as the key indicator for evaluation. For instance, Zhang et al [24] determined users' positive attitudes based on the number of likes a video received and found that content related to treatment, signs and symptoms, and social and cultural aspects in YouTube diabetes videos received the highest popularity. However, this approach often neglects the sentiment and emotions expressed in user comments, which offer valuable insights into viewers' genuine opinions. Analyzing user comments can provide a more comprehensive understanding of user preferences and help identify areas for improvement in video content creation and recommendation. To summarize, the informational quality of short videos on Douyin regarding oral diabetes medications is not well established, and research on user comment attitudes, as reflected in comments, is limited. Therefore, to bridge this gap, this study aimed to assess the information quality, content, and attitudes expressed in user comments on Douyin videos related to oral diabetes medications and explore the factors that influence these user comment attitudes.

## Methods

### Search Strategy and Data Extraction

This study conducted a basic search (simply entering the search term without additional filters) on the Douyin platform using the Chinese term “糖尿病口服药” (which means “oral diabetes medications”) on July 24, 2023, and selected the first 200 video web links delivered by Douyin's comprehensive ranking as our sample. The sample size was determined using the interval or ratio calculation formula [25], while considering the consistency levels of the DISCERN instrument (a tool for assessing health information quality) [26]. The calculated minimum sample size was 120, but considering the potential for invalid samples and referring to similar previous studies [17,18], 200 samples were ultimately determined. We chose to conduct the basic search on a single day to ensure consistency in the available video pool, given the platform's daily content updates. By using comprehensive ranking rather than focusing solely on the most popular or most recent videos, this method encompassed a broader range of content, potentially mitigating biases related to popularity or recency. Although search results may be influenced by Douyin's personalized recommendations for different users, this approach reflects typical user behavior when searching for health information. Notably, this sampling approach has been widely used in previous Douyin video analysis studies [17,19].

**Figure 1:**
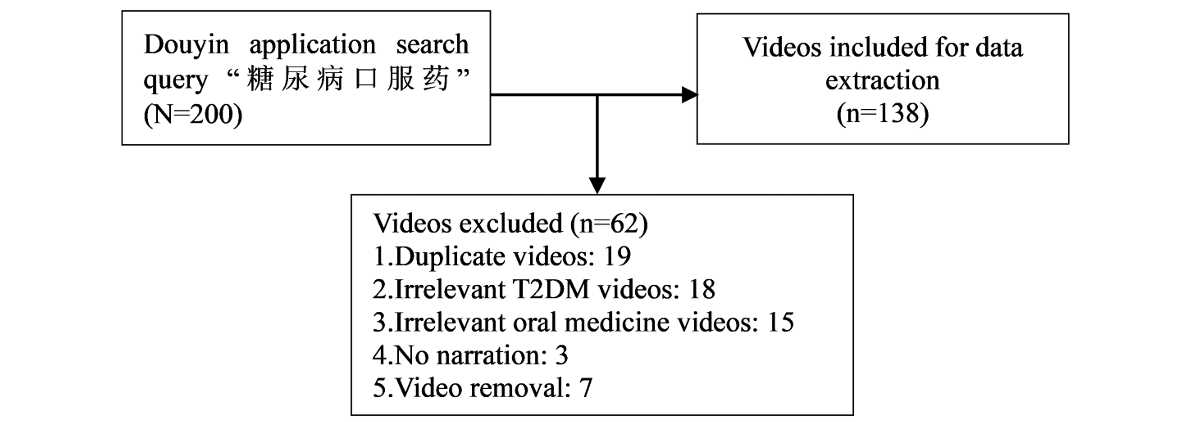
Flowchart of the screening process for T2DM-related videos on the Douyin platform from July 24 to 27, 2023. T2DM: type 2 diabetes mellitus.

All web links containing information from July 25-27, 2023, were extracted. Videos directly related to oral medicines for T2DM were included. Exclusion criteria were repeated videos; videos irrelevant to T2DM and oral medication; no narration, as narration is a key feature of educational videos that effectively delivers information through the combined use of audio and visual elements and videos lacking narration may not serve as comprehensive educational materials despite containing relevant visual information; and videos no longer available on the platform (taken off shelves), as their content and user comments could not be accessed for analysis. After the screening, we collected 138 (69%) of 200 videos for additional data extraction and analysis (Figure 1).

### Evaluation Methodology

Three aspects of oral diabetes medication-related videos on Douyin were evaluated: information quality, video content, and user comment attitudes. First, we used Python to extract video characteristics (days posted, length, number of likes, number of shares, user comments, and publisher replies to comments). Next, the main researcher manually reviewed the videos and associated publisher profiles on Douyin to collect publisher characteristics (source [doctor vs news agency], gender, department, and professional title). These characteristics were obtained from the publishers' self-reported information on their official account homepages. All this information was then organized in Microsoft Excel.

Second, we assessed the information quality using 2 instruments, the DISCERN and the PEMAT-A/V (Patient Education Materials Assessment Tool for Audiovisual Materials) [27]. DISCERN has been widely used to evaluate the quality of health information in Douyin short videos [17-19]. The instrument consists of 16 questions in 3 sections: reliability of the publication (questions 1-8), treatment choices (questions 9-15), and overall quality of the publication (question 16). Responses are rated using a 5-point Likert scale, ranging from 1 (poor) to 5 (good) [26]. PEMAT-A/V has been used as an instrument to evaluate the comprehensibility and actionability of patient education A/V materials [28]. The instrument consists of 17 questions in 2 sections: understandability (questions 1-13) and actionability (questions 14-17). The scores for the responses are 0 or 1, reflecting disagreement or agreement, respectively. The final score (%) equals (total points/total possible points) ×100. In PEMAT-A/V, a score of ≥70% in either understandability or actionability indicates that a particular aspect is considered good, while a score of <70% in any area means it is considered poor [28].

Third, we conducted content analysis and identified potential misinformation in the videos. The oral diabetes medication video content was classified into 9 aspects: mechanisms of action, indications, methods of administration, adverse effects, contraindications, efficacy, precautions, advantages, and importance based on the recent guidelines for oral hydrological medications [29] and the common themes identified by researchers during the review of video content. All 9 aspects were set as dichotomous variables (ie, mentioned vs not mentioned). The specific codebook is shown in Multimedia Appendix 1, which provides definitions and examples of each of the 9 aspects. During this content analysis process, the researchers also identified potential misinformation, which was defined as any content that contradicted or was inconsistent with the guidelines used for content classification. A third assessor (a Chinese endocrinology specialist) was consulted to verify the identified misinformation and proposed clarifications.

Furthermore, we implemented a rigorous rating process to ensure the reliability of our analysis. Two researchers independently worked on information quality assessment, content analysis, and identification of misinformation. Interrater reliability was evaluated using IBM SPSS Statistics 18.0 software. The interrater reliability ranges from 0.813 to 0.861 for 16 items in the DISCERN instrument, from 0.867 to 0.901 for 17 items in the PEMAT-A/V instrument, from 0.801 to 0.834 for each of the 9 aspects of medication video content, and from 0.811 to 0.821 for misinformation that pertains to video material. All reliability coefficients are statistically significant at the 0.1% error margin level. These results indicate satisfactory interrater reliability.

Finally, sentiment analysis of user comments was conducted using the Weiciyun website [30] to determine the proportion of attitudes (positive, neutral, and negative) for each video. The Weiciyun website, an online tool specialized in Chinese text analysis, leverages rule-based and machine learning algorithms to accurately identify sentiment tendencies in Chinese texts, while effectively handling context ambiguities. Compared to general sentiment attitude analysis tools, Weiciyun offers advantages in processing Chinese texts, incorporating advanced techniques for improved analysis quality [30]. Moreover, considering the large volume of comments to be analyzed, manual analysis would have been impractical and prone to human error. In contrast, the Weiciyun website enables efficient and code-free data analysis, often used in conjunction with Python, making it more suitable for this study.

The predominant attitude of video comments, determined by the highest percentage response (>50%), was used to categorize each video. The specific steps involved (1) importing user comments from each Python-crawled video; (2) incorporating custom sentiment words and modifying emoticon settings, such as adding words like “like,” “heart,” “applauding,” and “thanks” for positive sentiments and words like “sobbing,” “cracking up,” and “awkwardly laughing” for negative attitudes; (3) automatically analyzing the proportion of attitude in each video comment (positive, neutral, negative); and (4) assigning an attitude category based on the highest percentage. If in a video, no single category had a percentage >50% or the percentages were equally distributed among the 3 categories, the video was assigned a neutral attitude.

### Statistical Analysis

Data were analyzed using IBM SPSS Statistics 18.0 software. Categorical data were described as frequencies (%), while continuous variables were presented as the median (IQR) due to their nonnormal distribution. Chi-square tests and Kruskal-Wallis tests were performed for categorical and continuous variable comparisons, respectively, with Bonferroni adjustment for all pairwise comparisons. Variables with significant associations (*P*<.05) in these analyses were included in a multinomial logistic regression analysis to investigate the association between each video's basic characteristics, the quality of information (evaluated using DISCERN and PEMAT-A/V scores), and 9 content-related aspects with user comment attitudes.

### Ethical Considerations

This study analyzed publicly available videos on oral diabetes medications and their associated user comments on Douyin. All data collected were anonymized and deidentified. As the research did not involve direct human participation and only used public information, it was exempt from ethical review by the Mahidol University Institutional Review Board.

## Results

### Basic Characteristics and User Comment Attitudes in Videos

Table 1 shows the characteristics of oral diabetes medication videos on Douyin categorized by user comment attitude. In this study, 138 Douyin videos related to oral diabetes medications were analyzed. Regarding publisher characteristics, it was noted that most of these videos were uploaded by doctors on individual accounts (136/138, 98.6%). Conversely, the news agencies of organizational accounts contributed the least number of videos (2/138, 1.4%). The gender distribution of the publishers was relatively balanced, with males representing 54.3% (75/138) and females 45.7% (63/138). The majority of publishers were affiliated with the internal medicine department (115/138, 83.3%), while a smaller proportion were pharmacists (11/138, 8%). Most of the publishers held a senior professional title, serving as either the chief or deputy chief of their departments (113/138, 81.9%). In terms of video characteristics, the collected videos were posted on Douyin for a median duration of 217.5 (IQR 376) days. These videos were 70 (IQR 55) seconds long and received a median of 1897 (IQR 7957) likes, 385.5 (IQR 2400) shares, and 76.5 (IQR 242) comments. Notably, the median number of publisher replies was 0 (IQR 4). Regarding user comment attitudes, the majority of videos garnered positive comments (81/138, 58.7%), followed by neutral comments (46/138, 33.3%) and negative comments (11/138, 8%). The Kruskal-Wallis test results indicated significant differences in the number of user comments across videos with various comment attitudes (*P*=.002). Videos that received predominantly negative and neutral comments had significantly higher numbers of user comments compared to those that received predominantly positive comments (both *P*<.05).

**Table 1:** Characteristics of oral diabetes medication videos on Douyin categorized by user comment attitude.

Variables			Overall (N=138)		Positive comments (n=81)	Neutral comments (n=46)		Negative comments (n=11)		Statistical test	*P* value
**Publisher source, n (%)^a^**											
	Doctors	136 (98.6)		80 (98.8)		45 (97.8)	11 (100.0)		0.357		.840
	News agencies	2 (1.4)		1 (1.2)		1 (2.2)	0		—^b^		—
**Publisher gender, n (%)^a^**											
	Male	75 (54.3)		45 (55.6)		25 (54.3)	5 (45.5)		0.398		.820
	Female	63 (45.7)		36 (44.4)		21 (45.6)	6 (54.5)		—		—
**Publisher department, n (%)^a^**											
	Internal medicine	115 (83.3)		65 (80.2)		40 (87.0)	10 (90.9)		5.086		.530
	Surgery	6 (4.3)		4 (4.9)		2 (4.3)	0		—		—
	Pharmacy	11 (8.0)		6 (7.4)		4 (8.7)	1 (9.1)		—		—
	Others	6 (4.3)		6 (7.4)		0	0		—		—
**Publisher professional title, n (%)^a^**											
	Chief physician	80 (58.0)		43 (53.1)		30 (65.2)	7 (63.6)		2.757		.840
	Deputy chief physician	33 (23.9)		22 (27.2)		9 (19.6)	2 (18.2)		—		—
	Attending physician	14 (10.1)		10 (12.3)		3 (6.5)	1 (9.1)		—		—
	Pharmacist	11 (8.0)		6 (7.4)		4 (8.7)	1 (9.1)		—		—
**Video characteristics,** **median (IQR)^c^**											
	Days posted	217.5 (376)		250 (429)		196.5 (293)	173 (414)		0.868		.650
	Length (seconds)	70 (55)		71 (58)		70 (54)	59 (50)		0.101		.950
	Likes	1897 (7957)		1152(5511)		3724.5 (11461)	5623 (17578)		4.652		.100
	Shares	385.5 (2400)		216 (1491)		839 (6333)	2231 (7165)		5.594		.060
	User comments	76.5 (242)		38 (161)		150.5 (472)^d^	564 (698)^d^		12.707		.002
	Publisher replies with comments	0 (4)		0 (4)		0 (7)	0 (1)		1.468		.480

^a^Chi-square test.

^b^Not applicable.

^c^Kruskal-Wallis test.

^d^Significant differences from the positive-attitude group: neutral (*P*=.01) and negative (*P*=.03).

### Information Quality

Table 2 shows the information quality of oral diabetes medication videos on Douyin assessed by DISCERN and PEMAT-A/V and categorized by user comment attitude. Our findings indicated that the median overall information quality was 3 (IQR 1) out of a maximum score of 5 on the DISCERN instrument, and the median understandability was 75% (IQR 16.6%) for oral diabetes medication videos on Douyin. Both scores were considered acceptable. However, the median actionability was rated as poor, at 66.7% (IQR 48.0%). Kruskal-Wallis test results showed that the videos' reliability, information quality of treatment choices, total DISCERN scores, and PEMAT understandability scores exhibited significant differences across different user comment attitude types (*P*=.002, *P*=.001, *P*<.001, and *P*=.02, respectively). Furthermore, multiple comparison analysis showed that videos receiving predominantly positive comments had a significantly higher information quality of treatment choices and total DISCERN scores compared to those receiving neutral and negative comments and a significantly higher reliability and understandability compared to those receiving neutral comments (all *P*<.05).

**Table 2:** Information quality of oral diabetes medication videos on Douyin assessed by DISCERN and PEMAT-A/V^a^ and categorized by user comment attitude.

Variables			Overall (N=138), median (IQR)		Positive comments (n=81), median (IQR)	Neutral comments (n=46), median (IQR)		Negative comments (n=11), median (IQR)		Statistical test	*P* value
**DISCERN^b^**											
	Reliability of videos (questions 1-8)	25 (4)		26 (4)		24 (6)^c^	23 (4)		12.47		.002
	Information quality of treatment choices (questions 9-15)	15 (4)		16 (4)		14.50 (4)^c^	13 (3)^c^		13.42		.001
	Overall information quality (question 16)	3 (1)		3 (1)		3 (1)	3 (0)		4.19		.120
	Total DISCERN score	44 (6)		44 (5)		41 (8)^c^	40 (9)^c^		19.04		<.001
**PEMAT-A/V^b^**											
	Understandability	75% (16.6%)		77.8% (19.9%)		70% (18.6%)^c^	70% (16.6%)		7.59		.020
	Actionability	66.7% (48.0%)		66.7% (66.7%)		50% (33.4%)	66.7% (16.7%)		3.36		.190

^a^PEMAT-A/V: Patient Education Materials Assessment Tool for Audiovisual Materials.

^b^Kruskal-Wallis test.

^c^Significant differences from the positive-attitude group: reliability of videos (neutral *P*=.003); quality of treatment choices (neutral *P*=.006, negative *P*=.03); total DISCERN scores (neutral *P*<.001, negative *P*=.04); PEMAT-A/V understandability (neutral *P*=.03).

### Video Content

In terms of video content, Table 3 shows that among 138 videos, the mechanism of action was the most frequently mentioned theme with 75 (54.3%) videos, followed by precautions with 70 (50.7%) videos and advantages with 68 (49.3%) videos. In contrast, indications and contraindications were rarely mentioned, accounting for 19 (13.8%) and 14 (10.1%) videos, respectively. Moreover, chi-square test results demonstrated that there were statistically significant differences in the proportions of videos mentioning 2 content themes (mechanisms of action and advantages) across 3 types of user comment attitudes (*P*<.001 and *P*=.001, respectively). Additionally, multiple comparison analysis revealed that videos receiving predominantly positive comments mentioned these 2 themes at a significantly higher rate than those receiving neutral comments, and the difference was significant (*P*<.05). Regarding misinformation in video content, our results indicated that 10.1% (14/138) of the videos contained misinformation (Table 3). Furthermore, these 14 (10.1%) videos spanned 5 content themes (mechanisms of action, precautions, methods of administration, efficiency, and importance). It should be noted that the majority of the videos with misinformation (7/14, 50%) contained misinformation regarding methods of administration (Table 4).

**Table 3:** Content of oral diabetes medication videos on Douyin categorized by user comment attitude.

Variables			Overall (N=138), n (%)		Positive comments (n=81), n (%)	Neutral comments (n=46), n (%)		Negative comments (n=11), n (%)		Statistical test	*P* value
**Video content^a^**											
	Mechanism of action	75 (54.3)		55 (67.9)		14 (30.4)^b^	6 (54.5)		16.60		<.001
	Precautions	70 (50.7)		48 (59.3)		18 (39.1)	4 (36.4)		5.74		.060
	Advantages	68 (49.3)		30 (37.0)		33 (71.7)^b^	5 (45.5)		14.21		.001
	Efficacy	64 (46.4)		37 (45.7)		22 (47.8)	5 (45.5)		0.06		.970
	Methods of administration	54 (39.1)		34 (42.0)		17 (37.0)	3 (27.3)		1.02		.600
	Adverse effects	48 (34.8)		32 (39.5)		12 (26.1)	4 (36.4)		2.34		.310
	Importance	27 (19.6)		14 (17.3)		12 (26.1)	1 (9.1)		2.28		.320
	Indications	19 (13.8)		15 (18.5)		3 (6.5)	1 (9.1)		3.78		.150
	Contraindications	14 (10.1)		9 (11.1)		4 (8.7)	1 (9.1)		0.20		.900
Misinformation^a^			14 (10.1)		8 (9.9)	4 (8.7)		2 (18.2)		0.89	.640

^a^Chi-square test.

^b^Significant differences from the positive-attitude group: mechanisms of action (neutral *P*<.001); advantages (neutral *P*<.001).

**Table 4:** Misinformation and clarifications identified in oral diabetes medication videos on Douyin (N=14).

Content theme and misinformation			Videos, n (%)		Clarifications		
**Mechanisms of action**							
	Glinides are insulin sensitizers.	1 (7.1)		Erroneous. Glinides are insulinotropic agents.			
**Precautions**							
	Do not make up for a missed dose of glinide medicines after a meal.	2 (14.3)		Too absolute. Adjust the dose of glinide medicines between meals based on blood glucose levels; there is no need to make up the dosage if it is close to the next meal.			
**Methods of** **administration**							
	Metformin is taken with or after a meal.	6 (42.9)		Too general. Regular and extended-release metformin tablets are taken with and after meals, while enteric-coated tablets are taken before meals.			
	Take sulfonylurea medicines once or twice a day.	1 (7.1)		Too general. Take short-acting sulfonylurea medicines 3 times a day and medium- to long-acting ones once or twice daily.			
**Efficacy**							
	Glinides only control blood glucose levels after meals.	1 (7.1)		Too absolute. Glinides also have some effect on fasting blood glucose levels.			
	SGLT2^a^ inhibitors reduce postprandial blood glucose levels.	1 (7.1)		Too limited. SGLT2 inhibitors can reduce fasting and postprandial blood glucose levels.			
**Importance**							
	SGLT2 inhibitors and GLP-1^b^ receptor agonists have replaced insulin, sulfonylureas, and glargine as first-line hypoglycemic agents and should be chosen first.	1 (7.1)		Controversial.			
	Simeglutide can completely replace insulin.	1 (7.1)		Controversial.			

^a^SGLT2: sodium-glucose transporter 2.

^b^GLP-1: glucagon-like peptide-1.

### Factors Affecting User Comment Attitudes

Although the dependent variable (user comment attitudes: positive, neutral, negative) was ordinal, the independent variables did not pass the parallel line test (*P*<.001), prompting the use of multinomial logistic regression analysis in this study. Additionally, 10 independent variables from Tables 1, 2, and 3 with *P*<.05 were subjected to collinearity testing. It was found that except for “total DISCERN scores,” there was no collinearity among the remaining variables (tolerance>0.1 or variance inflation factor [VIF]<10). Consequently, 6 independent variables were ultimately included in the multinomial logistic regression analysis, and Table 5 presents the standardized results (*P*<.05).

**Table 5:** Multinomial logistic regression analysis results of factors associated with user comment attitudes toward oral diabetes medication videos on Douyin.

Dependent and independent variables		b (SE)		Wald	Adjusted OR^a^ (95% CI)		*P* value
**Positive comments^b^**							
	Comment count	–0.00 (0.00)	5.21		1.00 (1.00-1.00)	.020	
	Information quality of treatment choices	0.40 (0.16)	6.38		1.49 (1.09-2.04)	.010	
**Neutral comments^c^**							
	Information quality of treatment choices	–0.26 (0.10)	7.11		0.77 (0.64-0.93)	.010	
	Advantages (not mentioned)	–1.41 (0.48)	8.73		0.24 (0.10-0.62)	.003	
	Mechanism of action (not mentioned)	1.14 (0.46)	6.15		3.12 (1.27-7.66)	.010	
**Negative comments^b^**							
	Comment count	0.00 (0.00)	5.21		1.00 (1.00-1.00)	.020	
	Information quality of treatment choices	–0.40 (0.16)	6.38		0.67(0.49-0.91)	.010	

^a^OR: odds ratio.

^b^Using the negative category as a reference.

^c^Using the positive category as a reference.

In multinomial logistic regression analysis, influence factors, such as the number of user comments (*P*=.02) and the quality of information regarding treatment choices (*P*=.01), were found to be associated with videos receiving predominantly positive comments. However, videos receiving positive comments had an odds ratio (OR) of 1.00 (95% CI 1.00-1.00) for the number of user comments, indicating lower odds compared to videos receiving negative comments, which had an OR of 1.00 (95% CI 1.00-1.00). Furthermore, the OR for having high-quality information regarding treatment choices was 1.49 (95% CI 1.09-2.04) for videos receiving positive comments, while videos receiving negative comments had an OR of 0.67 (95% CI 0.49-0.91). However, videos receiving neutral comments had ORs of 0.77 (95% CI 0.64-0.93) for the information quality of treatment choices, 0.24 (95% CI 0.10-0.62) for the proportion of unmentioned medication advantages, and 3.12 (95% CI 1.27-7.66) for the proportion of unmentioned mechanisms of action (Table 5).

## Discussion

### Principal Findings

Our study explored the quality of information and user comment attitudes toward oral diabetes medication videos on Douyin. We found that most of the videos were uploaded by doctors, with generally acceptable information quality and received predominantly positive user comments. Notably, videos with higher information quality tended to receive more positive user comments. However, a minority of these videos contained misinformation, and the actionability of the video needs to be improved. Additionally, there was limited focus on the indications and contraindications of medications.

Our study determined that the quality of information in Douyin videos concerning oral medications for diabetes is generally acceptable [17]. This finding is similar to those for most other diseases, such as myopia [18] and chronic obstructive pulmonary disease [19], which were posted by health professionals. This may be ascribed to our findings that 98.6% of the videos on oral diabetes medications in our study originated from senior doctors' personal accounts. This was paired with a relatively low rate of misleading information, at only 10.9%, a notable contrast to other topics. For instance, YouTube videos on T2DM have a misleading information rate of 32.4% [31], while those on prostate cancer have a misleading information rate of 41.2% [32]; attention-deficit/hyperactivity disorder, 52% [33]; and pediatric urological diseases, as high as 77.8% [34]. Nevertheless, the occurrence of misinformation in videos disseminated by senior doctors underscores the necessity for continual improvement. Prior studies have confirmed that misleading online information can have harmful effects [35]. For instance, Weitzman et al [36] suggested that misleading information regarding diabetes cures prompts skepticism about their effectiveness. Therefore, our study suggests that health care professionals are crucial in maintaining the quality of health information, while also emphasizing the need to further ensure the accuracy of the health information they disseminate.

Unfortunately, the actionability of oral diabetes medication–related videos was found to be unsatisfactory. This lack of practical applicability is a concern, as patients often seek such videos for guidance on managing their health conditions. Our findings are not isolated; similar issues with actionability have been documented in online health information across various topics [32,33,37,38]. For instance, Xu et al [32] indicated that health videos on prostate cancer also frequently fall short in providing actionable steps. Zhang et al [39] further supported this, highlighting a general trend in online health content where the focus on providing clear, actionable advice is often lacking. These observations underline a critical gap in digital health communication, emphasizing the need for content creators, particularly health professionals, to prioritize actionability in their materials. As highlighted by Hilfiker et al [40], when using digital health information for decision-making, it is critical to focus on specific instructions and action steps. Therefore, we suggest that professor-level physicians contribute more patient-centered, high-quality videos on oral diabetes medication, emphasizing actionable content to provide patients with brief and clear instructions on necessary steps.

Regarding video content, our study noted that the majority of videos were about mechanisms of action, precautions, advancements, efficiency, and methods of administration, but only a few videos fully covered other aspects, such as indications and contraindications. Previous research has demonstrated the significant influence of digital health information on patients' decision-making [41]. When individuals search for information about diabetes medications, they often turn to online sources to determine the suitability of those medications for their condition and any potential contraindications. Consequently, a deficiency in clear and easily accessible information concerning indications and contraindications can result in misunderstandings and potentially harmful decisions. Furthermore, Douyin's official platform has recognized this issue and has required creators to specify indications and contraindications details when discussing treatments or medication [42]. Therefore, we suggest that health care professionals improve the provision of information concerning the indications and contraindications of oral diabetes medications to support patients in making well-informed health decisions.

This study suggested that the majority of user comments on oral diabetes medication–related videos on Douyin exhibit a positive comment attitude. In comparison to negative attitudes, the information quality of treatment choices in videos positively influenced users' attitudes. Previous studies have found that accurate, high-quality, well-structured, and informative content is essential for encouraging positive user engagement [43,44]. This study further revealed the importance of the information quality of treatment choices, including medication therapy principles, benefits and risks, consequences of nonuse, impact on individuals and families, and support for shared decision-making, positively influencing users' attitudes and reactions.

Moreover, an intriguing aspect of our findings is that the videos receiving predominantly positive comments were associated with significantly fewer user comments. The reason might be attributed to typical user behaviors and interaction patterns on social media, as well as the influence of individual personality differences. Prior studies have confirmed that users often prefer to observe and consume content silently on social media [45], and posts presented in video format or posts about rational content (informational, functional, and educational) do not significantly encourage active user comments [46,47]. Additionally, personality traits also affect online commenting behavior. Disagreeable individuals are more likely to comment to voice dissent, while neurotic users tend to comment more on emotionally impactful content [48]. Therefore, videos eliciting positive sentiment may suppress commenting driven by negative motivations (eg, disagreement or emotional distress) among these groups. Echoing these findings, our study revealed that users, upon viewing satisfying content regarding oral diabetes medication–related videos, consider it unnecessary to further comment.

It is worth mentioning that our study suggested there is no significant association between the length of the videos, the video duration, the publisher's gender, their professional title, and user comment attitudes toward Douyin videos related to oral diabetes medications. Previous studies on YouTube videos [24,49] have also confirmed that the video duration does not impact user comment attitudes. However, Zhang et al [24] found that the gender of publishers and the duration of posts significantly impact user comment attitudes toward diabetes-related video content on YouTube. This inconsistency may be attributed to the different video formats across platforms. Douyin videos are generally shorter than YouTube videos, requiring a shorter attention span, which might lead to different user engagement patterns. Our findings suggest focusing on the content and quality of oral diabetes medication–related videos on Douyin to help patients obtain accurate health information and promote medication adherence.

### Limitations and Future Directions

The findings of this study should be considered in the light of a few limitations. The videos analyzed in this study were in Chinese, which may limit the generalizability of the results to TikTok videos on oral diabetes medications in other languages. Future studies should assess the content quality of these videos in various languages for a more comprehensive understanding. Additionally, the selection of the top 200 videos recommended by Douyin's algorithm through a single-account search may introduce limitations, as search results can vary across user accounts due to personalized recommendations. Future research could explore multiaccount searches or longitudinal sampling to capture a broader content range and gain insights into the algorithm's functioning. Furthermore, the sentiment analysis conducted by the Weiciyun website may not fully capture the nuances and context of user comments due to language variability and algorithmic constraints, potentially leading to inaccuracies. It is recommended for future research to use more advanced sentiment analysis tools and algorithms to improve accuracy. Moreover, categorizing sentiment based on the highest percentage may oversimplify the distribution, particularly when sentiment is evenly spread across categories, leading to an incomplete representation of user comment attitudes. Future research could explore alternative methods for sentiment classification. Nonetheless, this study represents 1 of the first steps in advancing research involving the relationship between the information quality of Douyin oral diabetes medication videos and user comment attitudes, which goes beyond the previous studies limited to the broader spectrum of diabetes.

### Conclusion

Our study assessed the quality of information in 138 Douyin videos on oral diabetes medications. Results indicated that most videos, predominantly posted by doctors, generally offer acceptable information quality, focusing on mechanisms of action, precautions, and advantages. However, they lack comprehensive details on medication indications and contraindications, with some misinformation and dissatisfied actionability. User comment attitudes toward these videos are predominantly positive, influenced by the information quality of treatment choices. We conclude that patients should be cautious when seeking diabetes medication information on Douyin. We recommend that health care providers improve the accuracy and usefulness of their content to better inform treatment choices and enhance user confidence. Furthermore, we advise Douyin to refine its medical content review process.

## Appendix

Multimedia Appendix 1 Codebook for hand-coding oral diabetes medication-related Douyin videos.

## Abbreviations

A/V: audiovisual
GLP-1: glucagon-like peptide-1
OR: odds ratio
PEMAT-A/V: Patient Education Materials Assessment Tool for Audiovisual Materials
SGLT2: sodium-glucose transporter 2
T2DM: type 2 diabetes mellitus

## Data Availability

The data sets generated and analyzed during the study are not publicly available due to privacy concerns and content sensitivity. User comments and creator information pose identification risks, while health-related content about diabetes medications requires a proper context to avoid misinterpretation. The data sets are available from the corresponding author upon reasonable request.
