# Supplementary material for: Oral Diabetes Medication Videos on Douyin: Analysis of Information Quality and User Comment Attitudes
Source: JMIR Form Res. 2024 Oct 18;8:e57720. doi: 10.2196/57720 (PMC11530717; doi:10.2196/57720)
Supplement: Multimedia Appendix 1 [file formative_v8i1e57720_app1.docx]

**Multimedia Appendix 1**. Codebook for hand-coding oral diabetes medication-related Douyin videos.

| **Code** | **Definition** | **Examples** |
| --- | --- | --- |
| Mechanisms of action | Videos explaining how oral diabetes medications lower blood glucose through various processes in the body, such as improving insulin resistance, stimulating insulin secretion, or other specific mechanisms. | -Metformin reduces hepatic glucose output and improves peripheral insulin resistance.  -Sulfonylureas are insulin secretagogues that stimulate pancreatic β cells to secrete insulin and increase endogenous insulin levels.  -Repaglinide promotes insulin secretion to lower blood glucose levels. |
| Indications | Videos discuss specific conditions, patient groups, or clinical situations for which oral diabetes medications are prescribed or recommended. | -Metformin is used for diabetes, insulin resistance, overweight and obesity, lipid metabolism disorders, polycystic ovary syndrome, and cardiovascular protection.  -Gliclazide is suitable for T2DM patients with partially preserved pancreatic function, especially when diet and exercise alone are insufficient for adequate glycemic control. |
| Methods of administration | Videos detailing the specific ways to take oral diabetes medications, focusing on dosage forms, timing of administration, frequency, and dosage. | -Regular and extended-release metformin tablets should be taken with or after meals, while enteric-coated metformin tablets should be taken before meals.  -Sulfonylureas should be taken before meals. Short-acting sulfonylureas are taken three times a day, while medium- to long-acting ones are taken once or twice a day. |
| Adverse effects | Videos detailing potential side effects, risks, and negative outcomes of oral diabetes medications. | -Metformin may cause gastrointestinal reactions, vitamin B12 deficiency, and, in severe cases, lactic acidosis.  -Sulfonylureas can lead to hypoglycemia, weight gain, gastrointestinal reactions, skin reactions, liver damage, allergic reactions, and central nervous system symptoms. |
| Contraindications | Videos describing specific medical conditions, patient characteristics, or situations in which the use of certain oral diabetes medications is unsafe, potentially harmful, or not recommended. | -Metformin should not be used in patients with renal impairment, hepatic impairment, acute complications of diabetes, severe infection, heart failure, hypoxia, alcohol abuse, pregnancy and lactation, metformin allergy, and during procedures involving iodinated contrast media.  -Sulfonylureas are contraindicated in patients with Type 1 diabetes, diabetic ketoacidosis, severe hepatic or renal impairment, sulfonamide allergy, gestational diabetes, hyperosmolar hyperglycemic state, infection, trauma, major surgery, and emergency situations. |

**Multimedia Appendix 1.** Codebook for hand-coding diabetes oral medication-related Douyin videos (cont.)

| **Code** | **Definition** | **Examples** |
| --- | --- | --- |
| Efficacy | Videos mentioning the effectiveness of oral diabetes medications in lowering blood glucose, improving glycated hemoglobin (HbA1c), or other diabetes-related outcomes. | - Metformin lowers fasting and postprandial blood glucose, promotes weight loss, improves insulin sensitivity, and enhances cardiovascular outcomes.  - Sodium-glucose transporter 2 (SGLT2) inhibitors lower fasting and postprandial blood glucose and improve HbA1c.  -Glinides primarily reduce postprandial blood glucose, may affect fasting blood glucose, have a rapid onset and short duration of action, and improve HbA1c. |
| Precautions | Videos providing instructions on what to do if a dose of oral diabetes medication is missed, as well as other important safety considerations explicitly highlighted using cautionary language. For other precautions, see note 4. | -If a metformin dose is missed, take it as soon as possible. However, if it's close to the next scheduled dose, skip the missed dose and do not take a double dose.  - For glinide medications, adjust the dose between meals based on blood glucose levels. If a dose is missed and it's close to the next meal, there's no need to make up the dose. |
| Advantages | Videos highlighting the beneficial characteristics, positive effects, or distinctive features of specific oral diabetes medications or medication classes | -Metformin promotes weight loss, has a low risk of hypoglycemia, offers cardiovascular protective effects, and is low cost.  -DPP-4 inhibitors are less likely to cause hypoglycemia and weight gain.  -SGLT2 inhibitors have a low risk of hypoglycemia, contribute to weight loss, lower blood pressure, and provide cardio-renal protection. |
| Importance | Videos emphasizing the critical role, value, or significance of oral diabetes medications in managing diabetes. | - Metformin plays a crucial role in diabetes management as the first-line medication, offering a wide range of applications, safety, tolerability, preventive effects, and potentially additional health benefits, making it an essential tool in the treatment of diabetes.  - Sulfonylureas serve as essential alternative first-line or second-line medications in diabetes treatment, particularly when metformin is insufficient or not tolerated. |

Notes:

1. Mentioned" means the video touches upon any point related to the code's definition.

2. "Not mentioned" means the video does not cover any essential points of the code's definition.

3. The codebook serves as a guideline for coding various aspects of oral medications and does not require verbatim alignment. It suffices if the video captures the essence of the listed points.

4. Coding principles for other precautions: (1) Keyword identification: Priority should be given to content in the video that explicitly uses keywords such as "note", "should", "pay attention to", "be vigilant", "be cautious", "remember" and similar terms. (2) Content assessment: a) If the video mentions "pay attention to supplementing vitamin B12", code it as a precaution. b) If it only mentions "may cause vitamin B12 deficiency", then code it as an adverse reaction.
